# Health-Related Quality of Life of Rural Clients Seeking Telepsychology Services

**DOI:** 10.1155/2014/168158

**Published:** 2014-11-19

**Authors:** Kevin R. Tarlow, Carly E. McCord, Timothy R. Elliott, Daniel F. Brossart

**Affiliations:** ^1^Educational Psychology, Texas A&M University, College Station, TX 77843, USA; ^2^School of Public Health, Texas A&M Health Science Center, 8441 Highway 47, Clinical Building 1, Suite 1300, Bryan, TX 77807, USA

## Abstract

Sixty million US residents live in rural areas, but health policies and interventions developed from an urban mindset often fail to address the significant barriers to health experienced by these local communities. Telepsychology, or psychological services delivered by distance via technology, is an emerging treatment modality with special implications for underserved rural areas. This study found that a sample of rural residents seeking telepsychology services (*n* = 94) had low health-related quality of life (HRQOL), often due to cooccurring physical and mental health diagnoses including high rates of depression. However, a brief telepsychology treatment delivered to rural clients (*n* = 40) was associated with an improvement in mental health-related quality of life (*d* = 0.70, *P* < .001). These results indicate that despite the complex health needs of these underserved communities, telepsychology interventions may help offset the disparities in health service access in rural areas.

## 1. Introduction

Over 3.8 million of the 60 million rural United States residents live in Texas, which has more rural residents than any state in the country [1]. Approximately one in four rural residents has a mental illness or substance abuse problem [2]. However, the resources available to individuals living in rural areas are not equivalent to those available to their urban counterparts due to barriers such as lack of transportation, geographic isolation, low socioeconomic status, low educational attainment, and low rates of insurance coverage [3]. For instance, even though some mental health diagnoses occur at least as often in rural areas as in urban ones [4, 5], the negative impact of those diagnoses on health and quality of life may be greater in rural areas because of the barriers to availability, accessibility, and perceived stigma of mental health care services [6]. Rural-urban health disparities have been reported in groups ranging from clergy [7] to veterans [8].

Not only are rural residents confronted with unique barriers to service, but health care providers are less likely to deliver services in rural areas due to lower wages and compensation, increased ethical risk, and higher burn-out rates [9]. Given the range of rural health needs and the scarcity of specialty services, providers who do offer services in rural communities are often pressured to treat patients at the limits of their knowledge and expertise [10]. The unique features of the rural health care landscape require health policies, legislation, and interventions informed by these issues.

*Telehealth*, or the use of telecommunication technologies to provide health care services at a distance, is one way to offset the disparities in health care access found in rural areas. Telehealth describes a broad range of health-related services that may include use of internet, telephone, videoconferencing, email, chat, text messaging, and other technologies to improve access, affordability, and efficiency of service delivery. The American Psychological Association (APA) recently recognized the benefits of using* telepsychology* (i.e., psychological telehealth services) to increase access to underserved areas [11]. For the purposes of this paper, the term telepsychology is used to describe the delivery of individual psychotherapy via secure videoconferencing technology. In their guidelines for the use of this emerging treatment modality, APA suggested that health service providers should utilize telepsychology when research evidence supports its use for a particular client or population (p. 8). However, APA also pointed out that outcomes research for telepsychology may be lacking for many clients due to the developing nature of the field. The purpose of this paper is to (a) better understand the health needs of rural clients seeking mental health care services and (b) establish an evidence basis for telepsychology's effectiveness with rural clients. Meeting these two goals is an important step to increasing mental health care access for underserved rural areas.

A major challenge to providing health care services to rural communities—telehealth or otherwise—is the diversity of the rural communities themselves. As sociologist Daryl Hobbs said, “When you have seen* one *rural community, you have seen* one* rural community” (emphasis in original, p. 397) [12]. Rural health policies and interventions should be empirically and* locally* informed, based on the needs and resources of each unique community. Creating sustainable initiatives to meet health needs in rural areas often requires local collaboration among community stakeholders, including clinicians, researchers, students, community leaders, and other policy makers [13].

Understanding a rural community's available health resources is a vital first step in locally informed policy and intervention development.* Community capacity* describes a community's ability to mobilize its social, political, and organizational capital to meet unmet needs [14]. This begins with an assessment of needs and is followed by the development of deliberate, collaborative intervention strategies. The “build it and they will come” mentality will not lead to sustainability in rural areas. Universities may be powerful facilitators of community capacity, but they must collaborate with local stakeholders and maintain those relationships beyond their inception [15].

In 2006, the Center for Community Health Development (CCHD) at the Texas A&M Health Science Center (http://www.cchd.us/) conducted a survey in the Brazos Valley—a region of Texas comprised of seven counties, all of which are federally designated Health Professional Shortage Areas (HPSAs). Texas has the highest percentage of HPSAs in the country [16]. The 2006 survey found that mental health and public transportation were among the top needs of the region. Over half of respondents reported having at least one day of “poor mental health” in the last month, and 62% of respondents reported needing mental health services and being unable to get the needed services [17].

In response to the identified health needs of the area, a “town and gown” partnership was created between Leon County (one of the seven Brazos Valley counties) and Texas A&M University to provide telepsychology services to the area. A locally appointed health resource commission mobilized resources in Leon County to provide a physical location to conduct the telepsychology services, and in 2009 advanced doctoral level students in the Counseling Psychology program at Texas A&M University began providing long-distance services under the supervision of a licensed psychologist [18].

It was expected that many Leon County residents seeking telepsychology services would present with cooccurring physical and mental health conditions. Consequently, assessment of client needs, issues, and response to telepsychology treatment required an evaluation of client health-related quality of life (HRQOL). HRQOL is a multidimensional construct of well-being often encompassing physical, mental, emotional, and social functioning [19, 20]. The Centers for Disease Control and Prevention (CDC) suggest that measures such as the Medical Outcomes Study Short Form (SF-12) [21] can be used to identify needed services, inform policy and legislation, and evaluate outcomes of health interventions [22].

Three other studies of similar size have evaluated the impact of telepsychology and telemedicine for mental health treatment on HRQOL using the SF-12v2. In one Canadian study, telepsychiatry outperformed in-person psychiatry for mental health improvement [23]. In two European studies that included German-speaking clients in The Netherlands, Germany, Austria, and Switzerland, internet-based cognitive behavioral therapy (CBT) interventions for grief and posttraumatic stress disorder (PTSD) outperformed the waitlist control group for mental health improvement [24, 25]. While all three studies found moderate to large effects of telehealth interventions on mental health status, no effects on physical health status were detected.

The current study was developed with two goals. First, given the barriers to health experienced by many rural communities, we wish to better understand the physical and mental health status of rural residents receiving telepsychology services. We compare the physical and mental health scores for participants in the current study with established HRQOL norms for the overall US population and with a national sample of depressed individuals. We expect that participants presenting for telepsychology services will have a lower HRQOL than both the national standardization sample and the national sample of depressed individuals. We also briefly review the depressive symptoms reported by participants in order to better describe the clinical features of the sample.

Second, we examine the possible impact of telepsychology services on client HRQOL in this underserved area. We expect that participants who received four sessions of telepsychology services will demonstrate significant improvements in mental health status. However, we were uncertain of the degree to which telepsychology services would affect physical health functioning.

These two goals are independent but related:* What are the physical and mental health needs of this population? And given barriers to other health services, is telepsychology an effective treatment option for this group?* Answering these questions is essential for empirically and locally informed health policy development. We expect that a range of stakeholders in and outside of the Brazos Valley would benefit from a better understanding of (a) the HRQOL of this rural community and (b) the increased health service options made possible by emerging areas such as telepsychology.

## 2. Methods

### 2.1. Participants

Participants in the current study resided in Leon County, Texas, and received telepsychology services between 2009 and 2012. Leon County is a federally designated Health Professional Shortage Area (HPSA) for both medical and mental health services [16]. Participants were predominantly low-income and were treated for a variety of presenting concerns, including depression, anxiety, bipolar disorder, posttraumatic stress disorder (PTSD), and relationship concerns.

Two samples of participants were analyzed for this study. Participants in a larger sample (*n* = 94) completed self-report measures of physical and mental health (SF-12v2; the Patient Health Questionnaire, PHQ-9) [26] during their first telepsychology session, and those data were used in the first part of the study to make descriptive comparisons with nationally representative samples. Within those 94 participants, a smaller group (*n* = 40) was reassessed with the SF-12v2 at the fourth session. Those follow-up data were used in the second part of the current study to evaluate the effectiveness of telepsychology.

There were several reasons that 54 individuals in the larger sample did not participate in the follow-up portion of the study. In some cases, participants dropped out of treatment before the fourth session (*n* = 37). Clients dropped out of treatment for a variety of known and unknown reasons. Some clients discontinued services because their presenting concerns were met before the fourth session. Others dropped out because they lacked transportation, found other treatment providers, moved from the area, or were incarcerated. In some cases participants continued to receive treatment; however their assigned counselor did not participate in the follow-up study (*n* = 17) and SF-12 data were not collected at the fourth session. No inclusion or exclusion criteria were implemented in selecting clients for the follow-up study; rather the follow-up group counselors elected to readminister the SF-12. Supporting analyses not reported here found that the follow-up group (*n* = 40) and the non-follow-up group (*n* = 54) did not have statistically significant differences on their initial levels of physical health (PCS), mental health (MCS), or depression (PHQ-9), and the two groups were demographically similar. Based on these analyses, the 54 individuals not assessed for the follow-up group are not believed to represent a qualitatively different population of individuals, as their physical health, mental health, and demographic attributes were not different.

For the larger sample (*n* = 94), participants ranged in age from 14 to 73 years (*M* = 41.3, SD = 13.6). There were 71 women (75.5%) and 23 men (24.5%). The sample reflected the racial and ethnic composition of the county [27], with 71 white (75.5%), 5 black or African American (5.3%), 6 Hispanic or Latino (6.4%), 1 Asian (1.1%), and 1 biracial participants (1.1%), with 10 participants (10.6%) not reporting a race or ethnicity.

For the smaller sample (*n* = 40), participants ranged in age from 14 to 62 years (*M* = 40.6, SD = 13.2). There were 34 women (85.0%) and 6 men (15.0%). There were 34 white (85.0%), 2 black or African American (5.0%), 2 Hispanic or Latino (5.0%), 1 Asian (2.5%), and 1 biracial participants (2.5%).

### 2.2. Procedures

Clients were either self-referred or referred by a local physician to the telepsychology clinic. Initial phone screenings were conducted to screen out dangerous suicidal or homicidal ideation/intent and active psychosis. Clients were then seen in an intake interview to determine the presenting problem and to develop a treatment plan. All clients were verbally administered several assessments during the intake interview, including the SF-12v2. No clients were intentionally excluded from data collection, as the measures are part of normal clinic protocol. The SF-12v2 was verbally administered again during the fourth session to assess response to treatment.

Counseling sessions were approximately 50 minutes long and conducted via videoconferencing technology. Both the client and counselor sit in front of a 42-inch high definition television screen with at secure T1 connection meeting HIPAA guidelines for encryption and confidentiality. Counselors were doctoral level students in the Counseling Psychology program at Texas A&M University. Students were trained in a scientist-practitioner model and encouraged to use evidence-based treatments with clients. Common therapeutic approaches used by counselors include cognitive behavioral therapy, cognitive processing therapy, acceptance and commitment therapy, and other humanistic approaches to address the presenting and emerging problems identified by clients during the course of therapy.

### 2.3. Measures

#### 2.3.1. SF-12v2

The SF-12 [28] is a 12-item, self-report measure selected to measure health-related quality of life. A valid shorter version of the SF-36, the second edition of the SF-12 reproduces the total and composite scores with at least 90% accuracy. It is one of the most widely used health surveys in the world due to its proven practicality, utility, and psychometric stability [21]. The SF-12v2 yields two composite scores, the Physical Component Summary score (PCS) and the Mental Component Summary score (MCS), which reflect the overall level of physical and mental health status, respectively. Higher PCS scores indicate a higher level of physical functioning, and higher MCS scores indicate a higher level of mental and emotional functioning. PCS scores have an internal reliability coefficient of *α* = .89, and MCS scores have an internal reliability coefficient of *α* = .86. The two composite scores have strong statistical power, or the ability to detect true differences in health status when they occur [21].

The SF-12 includes norms so that individual and group scores can be compared to other relevant groups. The norming data was collected in 1998 from 7,069 individuals. Households were selected to match US census data on geographical region, market size, age, income, and household size. Scores are reported as standard T-scores with a mean of 50 and standard deviation of 10. However, other means and standard deviations have been calculated for specific groups. Some of the norms include a sample representative of the noninstitutionalized US population and subgroups based on gender, age, and common chronic health conditions such as diabetes, hypertension, depression, and arthritis [21].

#### 2.3.2. PHQ-9

The brief Patient Health Questionnaire [26] was administered at intake to assess depressive symptomology. Each item on the assessment corresponds to a single criterion from the* Diagnostic and Statistical Manual of Mental Disorders, Fourth Edition*. The items are scored on a Likert scale with the following options: 0 = not at all, 1 = several days, 2 = more than half the days, and 3 = nearly every day. Total scores range from 0 to 27. A total score of 10 or greater is a widely used cut-off score used to indicate a probable major depressive disorder diagnosis [29]. Scores can be interpreted based on the total score as well to classify severity: 0–4 indicates no depression; 5–9 mild depression; 10–14 moderate depression, 15–19 moderately severe depression, and greater than or equal to 20 severe depression. PHQ-9 scores have demonstrated stable psychometric properties with a test-retest coefficient of .84 and internal consistency estimates of *α* = .86 and .89 [26].

### 2.4. Analyses

To assess the relative health status of the present study's clinical sample (as measured by the SF-12v2), physical (PCS) and mental (MCS) health status data for participants in the current study are compared with a national standardization sample and with a national sample of depressed individuals using one-way ANOVA with Bonferroni post hoc comparisons. Data are also presented indicating the proportion of participants who met criteria for depressive symptomology (as measured by cut-off scores on the PHQ-9).

Analytic methods consistent with similar outcomes research [23–25] were used to determine the efficacy of telepsychology for participants in the current study. Paired *t*-tests were used to examine the statistical significance of physical and mental health status improvement after four sessions of telepsychology services. The effect size of treatment was calculated with Cohen's *d* for repeated measures [30].

## 3. Results

### 3.1. Sample Description and Normative Comparisons

*Health Status. *SF-12v2 data for clients are presented in Table 1 along with norms for a nationally representative sample of depressed individuals and norms for the general US population. A one-way ANOVA indicated that the samples in Table 1 had statistically significant differences for physical health status; *F*(2, 8781) = 69.44, *P* < .001. Bonferroni post hoc comparisons using Welch's *t*-tests indicated that the PCS scores for the rural telepsychology sample were not significantly different than the national depressed sample; *t*(104) = − 0.81, ns. However, the telepsychology sample had lower PCS scores than the national standardization sample; *t*(93) = −3.31, *P* = .001. Thus, the rural telepsychology sample had a lower physical health status than the nationally representative sample of the overall US population, whereas their physical health status was similar to a national sample of depressed individuals.

A second one-way ANOVA indicated that the samples in Table 1 had statistically significant differences for mental health status; *F*(2, 8790) = 844.12, *P* < .001. Bonferroni post hoc comparisons using Welch's *t*-tests indicated that the rural telepsychology sample had lower MCS scores than both the national standardization sample, *t*(94) = − 14.54, *P* < .001, and the national depressed sample, *t*(106) = − 4.61, *P* < .001.

Not only do the participants in the current study have poorer mental health status on average than a national sample of depressed individuals, but they occupy the extreme low end of the distribution of mental health status for the overall population. The average participant in the Leon County sample (MCS = 30.72) falls about two standard deviations below the mean of mental health status for the US population, well below the average individual.

*Depression. *Seventy-nine participants in the current study (84.0%) scored a 10 or greater on the PHQ-9, which is the most commonly used cut-off score for diagnosing major depressive disorder [29]. Thirty-eight participants (40.4%) scored at or above 20 on the PHQ-9, indicating a severe level of depression. Forty participants (42.6%) reported suicidal thoughts or thoughts of self-harm occurring for at least several days in the two weeks previous to their initial telepsychology session.

### 3.2. Effect of Telepsychology on HRQOL

Receiving four sessions of telepsychology services was associated with a statistically significant improvement in MCS scores between intake (*M* = 31.50, SD = 12.81) and session-four follow-up (*M* = 40.23, SD = 14.83), *t*(39) = 4.43, *P* < .001. An effect size for the improvement in mental health status was calculated using Cohen's *d* for repeated measures, *d* = 0.70, a moderate sized effect [30]. There was not a statistically significant change in PCS scores between intake (*M* = 44.12, SD = 16.86) and follow-up (*M* = 40.80, SD = 14.64), *t*(39) = −1.94, *P* = .060. These results are summarized in Table 2.

Due to the small number of participants in the study and the small number of men (*n* = 6) and participants of color (*n* = 6), analyses to detect differential treatment response for gender and race/ethnicity lacked sufficient statistical power for any meaningful interpretation.

As expected, age was associated with lower physical health status. A post hoc analysis found that the age of participants in the follow-up group had a statistically significant correlation with the PCS score of clients at intake (*r* = −.395, *P* = .012), and a similar correlation was found between age and PCS score for the larger sample of 94 participants (*r* = −.375, *P* < .01). No statistically significant correlations between age and mental health status were found.

## 4. Discussion

This study found that the health-related quality of life (HRQOL) of rural Texas residents presenting for telepsychology services is indeed poor. While participants in this study reported physical health problems, their mental health status was exceptionally low, with the average participant's mental health status score falling about two standard deviations below the average for the US population. Individuals seeking telepsychology services had even lower mental health scores than a national sample of individuals diagnosed with major depressive disorder. This finding is consistent with other studies of rural and urban HRQOL differences [7, 8].

Providing psychological services to clients in a Health Professional Shortage Area (HPSA) poses several challenges. Individuals seeking treatment are expected to present with cooccurring physical and mental health diagnoses, as was the case in the current study's sample. Many clients in this study's sample reported a range of chronic health and disability issues in addition to psychological concerns such as depression, trauma, substance abuse, and anxiety. Even with access to telepsychology services, many clients (and their counselors) may be undertreated for their physical health concerns. The ongoing lack of care experienced in rural HPSAs has clear implications for the provision of telepsychology services.

Despite the complexity of their presenting physical and mental health concerns, we found that telepsychology was effective at improving the mental health status of clients who were assessed after four sessions of therapy. These results add to the growing evidence base for telepsychology, and they show that this emerging treatment modality may be effective specifically for rural residents living in an underserved area. Our findings may be of special importance to health care providers in rural areas who have been encouraged to utilize telepsychology when outcomes research supports its use for that particular population [11].

While working in rural areas has its challenges, it is not without opportunities. As was seen in this study, utilizing academic-community partnerships provides opportunities to train future professionals in innovative service delivery models. There is also the potential for more collaborative care for health care consumers. In urban areas, it is virtually impossible for all the health providers to be familiar with the dozens or even hundreds of other providers in the area. But due to the small number of providers in rural areas, providers have the ability to know the names and faces of all other providers in the community. If interorganizational communication is encouraged and expected, community capacity can be built and consumers can expect more integrated care [31].

Sixty-two percent of individuals surveyed in the rural Brazos Valley region of Texas report being unable to access needed mental health services [4]. Given the numerous barriers to health experienced by many rural communities, it is possible that millions of rural US residents desire mental health care but are unable to access it. This study demonstrates that telepsychology services developed through an academic-community partnership are a viable option for rural residents in need of effective mental health care. Telepsychology is an emerging area of telehealth and telemedicine with rapidly growing evidence for its effectiveness, and its high accessibility has special implications for rural communities.

Limitations of the current study include the relatively small sample size, although this is a necessary aspect of studying groups living in sparsely populated areas. Due to the small number of participants, subsequent analyses lacked the statistical power to measure possible effects of gender or race/ethnicity on treatment outcome. While the focus of this study was on the response to a brief treatment (four sessions), future studies should also investigate the impact of long term treatment, especially given the chronic nature of many rural clients' presenting concerns. Counselors in the current study did not conduct a comprehensive assessment of all physical and mental health needs of clients; while global measures of HRQOL were used for this analysis, future studies may wish to investigate the prevalence and impact of specific diagnoses on response to telepsychology treatment. No clients were excluded from the study as long as they did not meet clinical exclusion criteria (e.g., high suicidal or homicidal risk, active psychosis); however several individuals seeking telepsychology treatment were unable to receive services because of mobility restrictions. All sessions were conducted at the community health center in Leon County, and individuals without cars were unable to attend weekly sessions. While this is a limitation of this study's design, many telehealth and telemedicine interventions use technology to deliver services directly to patients' homes [32–34]. Telepsychology researchers may wish to study in-home services as well; however special considerations should be made to ensure client confidentiality and safety [11].

## Figures and Tables

**Table 1:** SF-12v2 norm comparisons.

	Current study		National depressed sample [21]		National standardization sample [21]	
	PCS	MCS	PCS	MCS	PCS	MCS
Mean	44.46	30.72	45.77	36.85	49.70	49.48
SD	15.30	12.46	12.10	10.82	9.93	9.87

25th percentile	31.13	21.87	36.76	28.45	44.39	43.79
50th percentile	46.45	27.99	47.06	36.79	53.28	52.01
75th percentile	56.48	37.74	56.08	44.92	56.61	56.70

Minimum	16.16	−0.54	10.62	5.73	6.59	5.73
Maximum	70.78	63.19	71.82	65.53	71.82	71.01

*N*	94	94	1,000	1,002	7,690	7,697

**Table 2:** HRQOL outcomes at fourth telepsychology session.

	Initial session	Fourth session	*P*	Effect size
PCS	44.12 (16.86)	40.80 (14.64)	ns	—
MCS	31.50 (12.81)	40.23 (14.83)	<.001	0.70

Note: *n* = 40.
